# Short- and Long-Term Mortality in Severely Injured Older Trauma Patients: A Retrospective Analysis

**DOI:** 10.3390/jcm14062064

**Published:** 2025-03-18

**Authors:** Silviya Ivanova, Elsa F. Hilverdink, Johannes D. Bastian, Dominik A. Jakob, Aristomenis K. Exadaktylos, Marius J. B. Keel, Joerg C. Schefold, Helen Anwander, Thomas Lustenberger

**Affiliations:** 1 Department of Orthopedic Surgery and Traumatology, Inselspital, Bern University Hospital, 3010 Bern, Switzerlandjohannes.bastian@insel.ch (J.D.B.); helen.anwander@insel.ch (H.A.); thomas.lustenberger@insel.ch (T.L.); 2 Department of Orthopedic Surgery, Hand Surgery and Traumatology, Triemli Hospital, Birmendorferstrasse 497, 8063 Zurich, Switzerland; 3 Department of Visceral Surgery, Lindenhofspital, Bremgartenstrasse 117, 3001 Bern, Switzerland; 4 Department of Emergency Medicine, Inselspital, Bern University Hospital, 3010 Bern, Switzerland; aristomenis.exadaktylos@insel.ch; 5 Trauma Center Hirslanden, Clinic Hirslanden Zurich, Medical School, University of Zurich, Witellikerstrasse 40, 8032 Zurich, Switzerland; 6 Department of Intensive Care Medicine, Inselspital, Bern University Hospital, 3010 Bern, Switzerland

**Keywords:** polytrauma, mortality, older patients, orthogeriatrics

## Abstract

**Background/Objectives**: Older trauma patients experience increased in-hospital mortality due to the physiological challenges associated with aging and injury severity. However, limited data exist on long-term mortality rates beyond hospital discharge, particularly among severely injured elderly trauma patients. Understanding these outcomes is essential for improving clinical management and rehabilitation strategies. The objective of this study was to evaluate cumulative mortality rates (in-hospital, 28-day, 1-year, 2-year, and 3-year) in older trauma patients with an Injury Severity Score (ISS) ≥ 16. Independent risk factors for 1-year mortality were also identified. **Methods**: This retrospective cohort study included all trauma patients aged ≥ 65 years with ISS ≥ 16 admitted to the Emergency Department of our level 1 trauma center between January 2017 and December 2022. Demographic characteristics, injury patterns (Abbreviated Injury Scale (AIS) scores, ISS), and mortality rates were collected from electronic health records. Patients were stratified into two age groups: 65–80 years and >80 years. Mortality rates were compared with those in the corresponding age groups in the general Swiss population. Statistical analysis included Kaplan–Meier survival curves and logistic regression for identifying risk factors associated with 1-year mortality. **Results**: A total of 1189 older trauma patients with a mean ISS of 24.3 ± 7.9 were included. The most common injury was severe head trauma (AIS head ≥ 3: 70.6%), followed by chest trauma (AIS chest ≥ 3: 28.2%) and extremity injuries (AIS extremity ≥ 3: 17.4%). The overall in-hospital mortality rate was 10.3%. Mortality rates at 28 days, 1 year, 2 years, and 3 years were 15.8%, 26.5%, 31.5%, and 36.3%, respectively. Age-stratified analysis showed significantly higher mortality rates in patients aged > 80 years compared to the 65- to 80-year group at all post-discharge time points (28-day: 22.6% vs. 11.9%, *p* < 0.001; 1-year: 39.9% vs. 18.8%, *p* < 0.001; 2-year: 46.5% vs. 22.8%, *p* < 0.001; 3-year: 56.4% vs. 24.9%, *p* < 0.001). Compared to the general Swiss population, we observed significantly higher mortality rates at all measured time points in elderly trauma patients, particularly in those aged over 80 years, with 1-year mortality rates of 39.9% vs. 10% in the general population and 3-year mortality rates of 56.4% vs. 30% in the general population. Independent risk factors for 1-year mortality included advanced age and severe head injury (AIS head ≥ 3, *p* < 0.001). **Conclusions**: Severely injured elderly trauma patients face high long-term mortality risks, with 1-year mortality rates reaching 26.5% overall and nearly 40% in patients aged > 80 years. These findings highlight the need for research on tailored, holistic management strategies, including comprehensive in-hospital care, specialized neurorehabilitation, and post-discharge follow-up programs to improve survival and functional recovery in this vulnerable population.

## 1. Introduction

Older adults constitute a substantial and growing segment of the global population, with the proportion of individuals aged ≥ 65 years projected to increase from 9.3% in 2020 to 16.0% by 2050, surpassing 1.5 billion worldwide [1]. This demographic shift portends a corresponding rise in the incidence of trauma among older adults, currently ranked the fifth-leading cause of mortality, with a significant allocation of healthcare resources directed towards those aged 65 years and older [2]. Older trauma patients commonly experience poorer clinical outcomes, increased healthcare utilization, and higher complication rates compared to younger patients [3]. Numerous studies have identified age and injury severity as independent predictors of mortality in polytrauma patients [4,5]. In-hospital mortality rates among older polytrauma patients vary considerably, ranging from 10% to 57.4% [6,7,8], with rates as high as 60.8% reported among those aged ≥ 85 years [9]. This variability highlights the heterogeneous outcomes observed in this vulnerable patient cohort. Although the impact of low-energy injuries, such as hip fractures, on long-term mortality in older adults is well-documented—with a five- to eightfold increased risk for all-cause mortality compared to their non-fractured peers of the same age [10]—data regarding out-of-hospital mortality rates following severe trauma in this age segment remain limited.

Recent studies have highlighted the complexity and variability of mortality outcomes among elderly polytrauma patients. Severe traumatic brain injuries (TBIs) and orthopedic injuries are frequently encountered among older polytrauma patients, significantly influencing both short-term and long-term mortality outcomes [7,9]. Additionally, elderly trauma patients often experience undertriage, leading to delays in receiving appropriate trauma care, which further elevates mortality risks and worsens long-term outcomes [6,7]. The presence of frailty, preexisting comorbidities, and the complexity of the injury itself underscore the need for geriatric-specific prognostic models, as conventional trauma scoring systems may inadequately capture the nuances influencing mortality in this demographic [6].

The COVID-19 pandemic notably altered trauma epidemiology, influencing both trauma admission trends and injury patterns. Recent analyses revealed significant reductions in trauma admissions during pandemic lockdown periods, with changes in injury severity, demographics, and hospitalization duration [11,12]. These findings underscore the importance of utilizing predictive analytics and advanced data modeling to enhance healthcare system responsiveness during periods of fluctuating trauma incidence. Such approaches can significantly improve clinical outcomes by ensuring timely, targeted interventions and optimized resource allocation [11,12].

Given these complexities, there remains an urgent need to better understand the specific determinants and trajectory of long-term outcomes following severe trauma among elderly patients. Improving this understanding can guide resource allocation, enhance initial management strategies, and inform the development of targeted rehabilitation interventions. Furthermore, it is crucial to explore the role of preexisting conditions, frailty, and the adequacy of rehabilitation in driving observed mortality differences, as these factors may significantly impact outcomes independently of injury severity alone.

Therefore, this study aimed to investigate long-term mortality rates up to three years post-trauma in older patients with an Injury Severity Score (ISS) ≥ 16. We hypothesized that severely injured elderly patients exhibit significantly higher mortality rates at 1-, 2-, and 3-year intervals after trauma compared to the general Swiss population of the corresponding age group.

## 2. Materials and Methods

This retrospective cohort study was conducted in accordance with the guidelines of the Declaration of Helsinki and did not require ethics approval (in consent with the local institutional ethics committee Berne, Switzerland—BASEC 2024-00232).

The study population comprised all patients aged 65 years or older who sustained acute trauma with an Injury Severity Score (ISS) ≥ 16 and were treated in the Emergency Department at the University Hospital of Bern (level 1 trauma center) between 1 January 2017 and 31 December 2022. Demographic data, initial vital signs, and injury mechanism and pattern (including ISS and Abbreviated Injury Scale (AIS) score for different body regions) were retrospectively extracted from the electronic patient records. Additional variables collected included sex, exact age at injury, date and time of hospital admission, duration of intensive care unit (ICU) stay, and overall hospital length of stay. In-hospital mortality was defined as death occurring from admission until hospital discharge, while 28-day mortality was defined as death within 28 days following trauma. Out-of-hospital mortality data were obtained through linkage with the national registry, enabling reliable tracking of patients’ survival status up to three years post-trauma. This approach allowed us to accurately assess post-discharge mortality rates at 1-, 2-, and 3-year intervals. Patients with acute-on-chronic subdural hematomas were explicitly excluded due to the chronic nature and distinct clinical trajectory of these injuries and potential for artificially inflated ISS values, which could have introduced substantial bias in the analysis of trauma-related mortality outcomes. Furthermore, patients without a documented “General Consent Obtained” status and foreign patients (due to a lack of follow-up data) were excluded.

The 1-year mortality rate was analyzed for the entire study group (*n* = 1189). The 2-year mortality rate was assessed for patients admitted between 1 January 2017 and 31 December 2020 (*n* = 985), while the 3-year mortality rate was calculated for the subgroup admitted between 1 January 2017 and 31 December 2019 (*n* = 818). These mortality rates were compared with corresponding rates from the general Swiss population within the same age groups. Data on national mortality rates were obtained from the Federal Statistical Office (www.bfs.admin.ch, accessed on 22 February 2024), which provides 1-, 2-, and 3-year mortality rates stratified by age group. Patients were categorized into two age groups: 65–80 years and >80 years at the time of trauma. Mortality rates were further stratified by ISS categories: ≥16 and ≥25 points.

The objective of this study was to evaluate both in-hospital and out-of-hospital mortality rates at 1, 2, and 3 years post-trauma. Independent risk factors associated with 1-year mortality in this cohort were also investigated.

### Statistical Analyses

Continuous variables are presented as means ± standard deviation (SD), while categorical variables are expressed as percentages. *p*-values for categorical variables were calculated using the chi-squared test or Fisher’s exact test as appropriate. Differences were considered statistically significant at *p* < 0.05. Survival analysis was conducted using Kaplan–Meier curves, which were compared using the log-rank test. To identify independent risk factors associated with 1-year mortality, a stepwise logistic regression model was applied. Variables with a *p*-value < 0.2 in the bivariate analysis were included in the multivariate model. All statistical analyses were performed using Statistical Package for Social Sciences (SPSS) software, version 24.0 (SPSS Inc., Chicago, IL, USA).

## 3. Results

During the study period, a total of 1670 patients aged ≥ 65 years with an ISS ≥ 16 were admitted to the Emergency Department of the University Hospital of Bern. Of these, 481 patients presented with acute-on-chronic subdural hematomas and were therefore excluded. The remaining 1189 patients constituted the study cohort and were further analyzed. The mean ISS was 24.3 ± 7.9, and the mean age of the patients was 77.7 ± 7.7 years (range 65–101 years). The majority of patients (*n* = 1173, 98.7%) sustained blunt trauma. Severe head injuries were the most frequently observed (70.6%), followed by severe chest injuries (28.2%) and lower-extremity injuries (16.6%). Table 1 outlines the demographic and injury characteristics, comparing the two study groups.

The overall in-hospital mortality rate was 10.3% (*n* = 123). The 28-day, 1-year, 2-year, and 3-year mortality rates were 15.8%, 26.5%, 31.5%, and 36.3%, respectively (Table 2).

**Figure 1:**
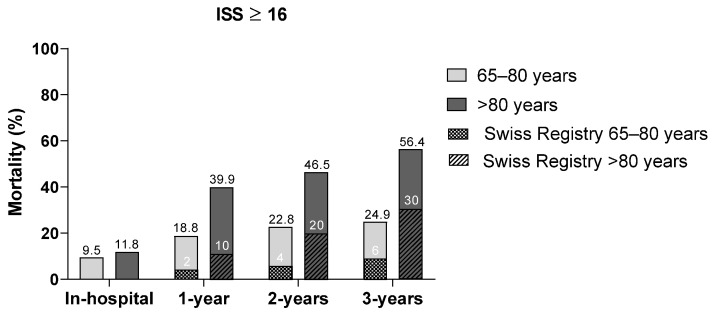
Comparison of overall mortality rates in patients aged 65–80 and >80 years with ISS ≥ 16 at various time points.

Figure 1 illustrates the overall mortality rates at different time points stratified by age group. Mortality rates at all time points were significantly higher in the age group >80 years compared to their younger counterparts. Furthermore, in both age groups, overall mortality rates among trauma patients were markedly higher than in the corresponding age segment of the general Swiss population. Post-discharge mortality within the first year was 18.8% in patients aged ≤ 80 years and 39.9% in patients >80 years (compared to 2% and 10% 1-year mortality rates, respectively, in the general Swiss population). However, in the second and third years after trauma, the difference in mortality rates between the study cohort and the general Swiss population diminished (e.g., >80 years, 2-year vs. 1-year mortality: 46.5%–39.9% = 6.6% vs. 20%–10% = 10% in the Swiss population).

**Figure 2:**
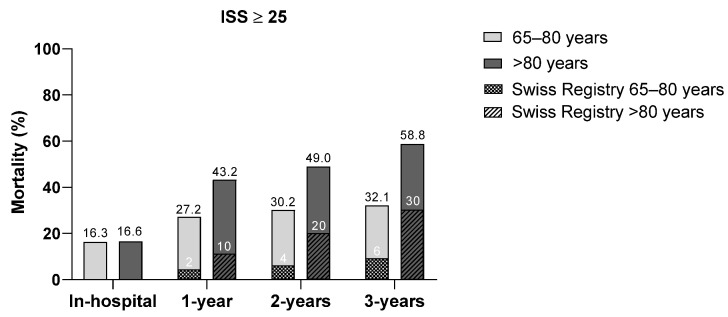
Comparison of post-injury mortality rates in patients aged 65–80 vs. >80 years with ISS ≥ 25 at various time points.

Figure 2 depicts the corresponding analysis for the subgroup of patients with an ISS ≥ 25. Similarly, mortality rates were significantly higher in patients >80 years compared to younger patients and were consistently higher than those in the general Swiss population. Post-discharge mortality in the first year (*n* = 640) was 27.2% for patients aged ≤80 years and 43.2% for those >80 years (vs. 1-year mortality rates of 2% and 10%, respectively, in the general Swiss population). In the second year after trauma (*n* = 540), the difference in mortality rates between the study cohort and the general Swiss population narrowed. However, in the third year after trauma (*n* = 450), the difference widened again (≤80 years: 32.1% vs. 2% in the Swiss population; >80 years: 58.8% vs. 10% in the Swiss population).

**Figure 3:**
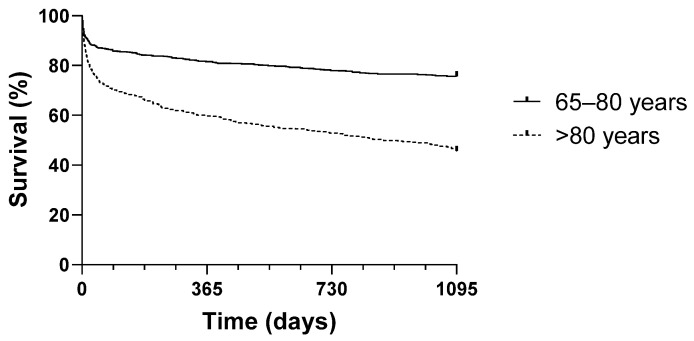
Kaplan–Meier survival curves for survival probability (intervals of 30 days, 6 months, and 1, 2, or 3 years) for individuals aged ≤80 years and those aged >80 years.

**Figure 4:**
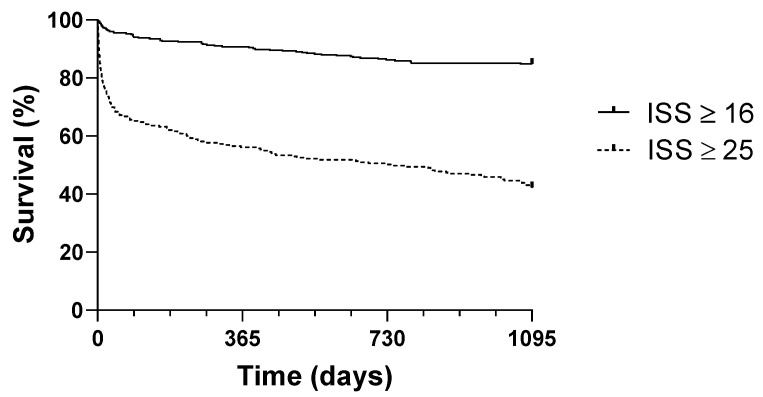
Kaplan–Meier survival curves for survival probability based on Injury Severity Score (ISS ≥ 16 and ISS ≥ 25).

Figure 3 and Figure 4 show significantly diverging Kaplan–Meier survival curves for the overall study group and the subgroup of patients with an ISS ≥ 25 (log-rank test, *p* < 0.001).

Bivariate analysis was performed to identify risk factors associated with in-hospital and 1-year mortality. Stepwise logistic regression analysis identified age, severe head and chest injuries, and overall ISS as independent risk factors for in-hospital mortality following trauma in elderly patients (Table 3). Among patients surviving hospital discharge, age and severe head injuries were independently associated with 1-year mortality.

## 4. Discussion

This study addresses a critical gap in understanding the long-term outcomes of older adults sustaining severe trauma, offering essential insights for optimizing clinical management strategies and improving survival rates within this increasingly significant demographic. We observed a 1-year mortality rate of 26.5% in the overall patient cohort, more than double the in-hospital mortality rate (10.3%). In patients aged > 80 years, the 1-year mortality rate approached 40%, corresponding to a 27% post-discharge mortality rate in the first year. In patients aged ≤ 80 years, the 1-year mortality rate was nearly 19%, reflecting a 10% post-discharge mortality rate in the first year after trauma. A similar pattern was observed in the subgroup of patients with ISS ≥ 25 points. Independent risk factors for 1-year mortality, both in the entire cohort and among those surviving hospital discharge, included age, overall injury severity, and significant head injuries. These findings emphasize the severe impact trauma has on long-term survival in older patients, particularly highlighting the increased vulnerability in patients aged over 80 years. Moreover, our results underscore the importance of comprehensive geriatric trauma care that addresses not only acute injury management but also longer-term outcomes and rehabilitation needs in this growing patient population. Given the rapid demographic shift towards an aging population, healthcare systems must adapt by developing targeted approaches for acute trauma care, incorporating geriatric expertise into trauma teams, and optimizing post-acute care pathways.

Overall, 70% of our study cohort sustained severe head trauma, with 75% of patients aged >80 years having an AIS head score ≥ 3. This aligns with the rising incidence of traumatic brain injury (TBI) among older adults [13,14]. Traumatic head injuries significantly impact long-term outcomes, partially explaining the high 1-year mortality rates observed in this study. Similarly, a recent study found that patients with a head AIS score of 3 experienced significantly higher rates of major complications, increased ICU admissions, and elevated in-hospital and 1-year mortality rates [15]. Importantly, even mild-to-moderate head injuries have been identified as significant predictors of mortality in elderly patients following ground-level falls [16]. In this study, severe head injuries emerged as significant independent risk factors for both in-hospital and 1-year post-discharge mortality. Additionally, the rate of discharge-to-rehabilitation programs was low, at 15.7%. This underscores the critical need for specialized neurorehabilitation for older trauma patients. A recent Swedish national cohort study found that most elderly patients did not receive rehabilitation from caregivers experienced in managing brain injuries [17]. Older age was negatively associated with access to specialized rehabilitation, consistent with previous studies and supported by our findings [18]. Rehabilitating trauma patients to restore independence is a substantial challenge. Recent evidence indicates that even patients hospitalized for mild TBI may experience disabling symptoms six months post-injury, including cognitive impairments, physical limitations, difficulties with daily activities, psychological issues, and speech problems [19]. Thus, the development of neurorehabilitation programs within healthcare systems significantly impacts long-term outcomes [20]. In Switzerland, high-level rehabilitation care is available for trauma victims, though potential inequalities related to age or socioeconomic status require continuous evaluation. Our findings suggest a potential unmet need for rehabilitation in elderly trauma patients. Therefore, future research should rigorously assess the direct impact of rehabilitation programs on reducing long-term mortality and improving functional independence, further clarifying the role of rehabilitation in enhancing post-discharge survival. The limitations of the study are as follows. The definition of geriatric polytrauma is under debate. The present study used the ISS to define severely injured older patients. However, the ISS has well-known limitations: it considers only the most severe injury in each body region, potentially overlooking additional serious injuries in the same region, and does not account for age as a criterion [21]. Alternative scores tailored for older patients have been proposed, such as the Geriatric Trauma Outcome Score (GTOS), which predicts in-hospital mortality based on ISS, age, and transfusion requirements at intensive care unit (ICU) admission [22]. Additionally, frailty may be more relevant than age alone. The GERtality Score [23], developed using data from the German Trauma Registry, includes five parameters: age ≥ 80 years, AIS ≥ 4, packed red blood cell (PRBC) transfusion before ICU admission, ASA (American Society of Anesthesiologists) score ≥ 3, and GCS (Glasgow Coma Scale) < 14 points. A maximum score of 5 corresponds to a 72.4% mortality rate. However, like the GTOS, this score focuses on short-term outcomes and incorporates the AIS. Since none of these scoring systems has been universally adopted in geriatric trauma research, we opted to use the ISS to define severely injured older patients, acknowledging its limitations.

Another limitation is the retrospective design of our study. Variables such as comorbidities and in-hospital complications were not available, and causes of out-of-hospital death were not documented. Specifically, the lack of detailed data on chronic conditions (e.g., diabetes, hypertension, COPD) and complications during hospitalization might have prevented us from identifying important confounding factors or risk modifiers. Furthermore, without documentation of the precise causes of death occurring after hospital discharge, it remains challenging to distinguish mortality directly attributable to trauma from that associated with chronic health conditions or unrelated medical events. Additionally, mortality following trauma differs fundamentally from other age-related conditions due to its acute onset, the rapid physiological deterioration, and trauma-specific complications such as systemic inflammatory response syndrome (SIRS), acute organ failure, or sepsis. These unique aspects of trauma necessitate different clinical management strategies compared to chronic age-related diseases. Due to these considerations, detailed analyses of competing mortality risks were not feasible, but represent an essential area for future research, explicitly addressing whether mortality differences among elderly trauma patients are primarily driven by trauma severity, preexisting comorbidities, frailty, or inadequate rehabilitation. Prospective studies incorporating standardized documentation of these parameters could significantly clarify the relative contributions of these factors. Implementing comprehensive data collection protocols in trauma centers, including frailty and comorbidity assessments, would enhance prognostic accuracy and allow for individualized care strategies.

The generalizability of our findings may also be limited due to variations in healthcare systems, care pathways, and the availability of specialized geriatric rehabilitation services across different institutions or countries. Nearly half of the patients were transferred to other hospitals following acute care, complicating the accurate interpretation of discharge status and possibly underestimating actual rehabilitation referrals. Additionally, using ISS and age as inclusion criteria created a clinically heterogeneous patient cohort, reflecting real-world scenarios, yet this limits the comparability with studies employing more precise geriatric-specific stratification methods. The ISS itself has recognized limitations, as it overlooks multiple significant injuries within the same body region and does not adequately incorporate the impact of age or preexisting conditions. Although frailty indices like the GERtality Score have demonstrated greater prognostic accuracy, especially for elderly populations, we could not utilize these due to dataset limitations. Chronic subdural hematomas were intentionally excluded to avoid artificially inflating injury severity scores and to minimize potential confounding effects from preexisting conditions.

Despite these limitations, our study has several notable strengths. Firstly, it included a large and well-defined cohort of elderly trauma patients with an ISS ≥ 16 from a major level 1 trauma center, providing robust data on both in-hospital and long-term outcomes. Additionally, the extended follow-up period of up to three years allowed a detailed evaluation of mortality trends over time, enhancing the understanding of trauma’s long-term impact on elderly patients beyond the acute hospitalization phase. Furthermore, comparing mortality rates with age-matched data from the general Swiss population provided a meaningful benchmark, clearly illustrating the excess mortality burden associated with severe trauma in older adults.

## 5. Conclusions

To the best of our knowledge, this is the largest study conducted in Switzerland investigating long-term mortality rates up to three years after trauma in older, severely injured patients. We found 1-year mortality rates of 19% in patients aged ≤80 years and 40% in those aged >80 years. However, by the second and third years after trauma, mortality rates approached those of the general Swiss population of the same age. Independent risk factors for 1-year post-discharge mortality included age, overall injury severity, and head injury, emphasizing the critical need for specialized rehabilitation programs, particularly neurorehabilitation, regardless of the patient’s age. These findings are crucial for refining clinical management strategies and enhancing survival outcomes in this increasingly significant demographic.

## Figures and Tables

**Table 1:** Demographics and injury characteristics stratified by patient age.

	All Patients (*n* = 1189)	65–80 Years (*n* = 755)	>80 Years (*n* = 434)	*p*-Value
Males, % (*n*)	61.9 (736)	67.0 (506)	53.0 (230)	<0.001
GCS, mean ± SD	13.0 ± 3.6	12.8 ± 3.8	13.4 ±3.1	<0.001
GCS ≤ 8, % (*n*)	10.7 (127)	12.6 (95)	7.4 (32)	0.005
ISS, mean ± SD	24.3 ± 7.9	24.4 ± 8.4	23.9 ± 6.9	0.004
ISS ≥ 25, % (*n*)	54.8 (651)	52.7 (398)	58.3 (253)	0.063
AIS head ≥ 3, % (*n*)	70.6 (839)	67.9 (513)	75.1 (326)	0.009
AIS thorax ≥ 3, % (*n*)	28.2 (335)	31.1 (235)	23 (100)	0.003
AIS abdomen ≥ 3, % (*n*)	5.6 (66)	6.4 (48)	4.1 (18)	0.109
AIS upper extremity ≥ 3, % (*n*)	1.2 (14)	1.5 (11)	0.7 (3)	0.239
AIS lower extremity ≥ 3, % (*n*)	16.6 (197)	17.1 (129)	15.7 (68)	0.527
Duration in intensive care (h)	58.7 ± 78	66.3 ± 98.4	90.4 ± 47.8	<0.001
Length of stay (d)	9.3 ± 13.7	10.1 ±15.5	8 ± 9.9	0.01
Injury mechanism				
Blunt trauma, % (*n*)	98.7 (1173)	98.3 (742)	99.3 (431)	0.279
Traffic accident, % (*n*)	22.5 (267)	27.5 (208)	13.7 (59)	<0.001
Fall at ground level, % (*n*)	13.5 (160)	10.5 (79)	18.7 (81)	<0.001
Fall < 3 m, % (*n*)	43.8 (521)	38.4 (290)	53.2 (231)	<0.001
Fall > 3 m, % (*n*)	11.2 (133)	13.9 (105)	6.5 (28)	<0.001

**Table 2:** Mortality and discharge status.

	All Patients (*n* = 1189)	65–80 Years (*n* = 755)	>80 Years (*n* = 434)	*p*-Value
Mortality				
In-hospital mortality, % (*n*)	10.3 (123)	9.5 (72)	11.8 (51)	0.227
1-year mortality, % (*n*)	26.5 (315)	18.8 (142)	39.9 (173)	<0.001
2-year mortality, % (*n*)	31.5 (310)	22.8 (143)	46.5 (167)	<0.001
3-year mortality, % (*n*)	36.3 (297)	24.9 (130)	56.4 (167)	<0.001
Discharge status				
Home, % (*n*)	19.2 (228)	24.0 (181)	10.8 (47)	<0.001
Elderly home, % (*n*)	5.8 (69)	4.0 (30)	9.0 (39)	<0.001
Rehabilitation, % (*n*)	15.7 (187)	18.4 (139)	11.1 (48)	<0.001
Other hospital, % (*n*)	48.2 (573)	43.3 (327)	56.7 (246)	<0.001
Other/unknown, % (*n*)	0.6 (7)	0.7 (5)	0.5 (2)	1.0

**Table 3:** Independent risk factors for 1-year mortality.

Risk Factors for In-Hospital Mortality in All Patients	*p*-Value	Adjusted OR (95% CI)
Age	0.001	1.05 (1.02–1.08)
AIS head ≥ 3	<0.001	5.98 (3.71–9.63)
ISS	<0.001	1.12 (1.09–1.16)
Risk Factors for 1-Year Mortality in Patients Surviving Hospital Discharge		
Age	<0.001	1.12 (1.09–1.15)
AIS head ≥ 3	0.001	2.59 (1.46–4.58)

## Data Availability

The data presented in this study are available on request from the corresponding author.

## Abbreviations

The following abbreviations are used in this manuscript:

ISS	Injury Severity Score
AIS	Abbreviated Injury Scale
SPSS	Statistical Package for Social Sciences
GTOS	Geriatric Trauma Outcome Score
ICU	intensive care unit
PRBCs	packed red blood cells
TBI	traumatic brain injury
